# Kind Discrimination and Competitive Exclusion Mediated by Contact-Dependent Growth Inhibition Systems Shape Biofilm Community Structure

**DOI:** 10.1371/journal.ppat.1004076

**Published:** 2014-04-17

**Authors:** Melissa S. Anderson, Erin C. Garcia, Peggy A. Cotter

**Affiliations:** Department of Microbiology & Immunology, School of Medicine, University of North Carolina at Chapel Hill, Chapel Hill, North Carolina, United States of America; University of Washington, United States of America

## Abstract

Contact-Dependent Growth Inhibition (CDI) is a phenomenon in which bacteria use the toxic C-terminus of a large exoprotein (called BcpA in *Burkholderia* species) to inhibit the growth of neighboring bacteria upon cell-cell contact. CDI systems are present in a wide range of Gram-negative proteobacteria and a hallmark feature is polymorphism amongst the exoprotein C-termini (BcpA-CT in *Burkholderia*) and amongst the small immunity proteins (BcpI) that protect against CDI in an allele-specific manner. In addition to CDI, the BcpAIOB proteins of *Burkholderia thailandensis* mediate biofilm formation, and they do so independent of BcpA-mediated interbacterial competition, suggesting a cooperative role for CDI system proteins in this process. CDI has previously only been demonstrated between CDI^+^ and CDI^−^ bacteria, leaving the roles of CDI system-mediated interbacterial competition and of CDI system diversity in nature unknown. We constructed *B. thailandensis* strains that differed only in the BcpA-CT and BcpI proteins they produced. When co-cultured on agar, these strains each participated in CDI and the outcome of the competition depended on both CDI system efficiency and relative bacterial numbers initially. Strains also participated in CDI during biofilm development, resulting in pillar structures that were composed of only a single BcpA-CT/BcpI type. Moreover, a strain producing BcpA-CT/BcpI proteins of one type was prevented from joining a pre-established biofilm community composed of bacteria producing BcpA-CT/BcpI proteins of a different type, unless it also produced the BcpI protein of the established strain. Bacteria can therefore use CDI systems for kind recognition and competitive exclusion of ‘non-self’ bacteria from a pre-established biofilm. Our data indicate that CDI systems function in both cooperative and competitive behaviors to build microbial communities that are composed of only bacteria that are related via their CDI system alleles.

## Author Summary

Contact-Dependent Growth Inhibition (CDI) systems are highly diverse interbacterial competition systems that bacteria use to kill neighboring bacteria upon cell-cell contact. In *Burkholderia* species, BcpA is the large exoprotein responsible for mediating CDI. BcpI proteins provide immunity against auto-inhibition. Diversity of CDI systems exists within the toxic C-terminus of BcpA proteins (called the BcpA-CT) and BcpI proteins. In addition to mediating interbacterial competition in *Burkholderia thailandensis*, BcpA also mediates biofilm formation, suggesting CDI system proteins play a cooperative role in nature. However, the roles of CDI system-mediated interbacterial competition and of CDI system diversity in nature are unclear. We constructed *B. thailandensis* strains that produced different BcpA-CT and BcpI proteins. Bacteria participated in CDI during biofilm formation, resulting in biofilm structures that were segregated by CDI system protein types. Furthermore, competition via CDI allowed bacteria in a pre-established biofilm community producing one set of CDI system proteins to exclude bacteria producing a different set of CDI system proteins from entering the community. Our data imply, therefore, that CDI-mediated competition and CDI system diversity function as a mechanism for self-recognition during the development of microbial communities.

## Introduction

Contact Dependent Growth Inhibition (CDI) is a phenomenon discovered in *E. coli* as a mechanism for interbacterial competition; *E. coli* producing the Two Partner Secretion (TPS) pathway proteins CdiA (a TpsA-family exoprotein) and CdiB (a TpsB-family outer membrane channel protein) inhibit the growth of specific CDI^−^ *E. coli* upon cell-cell contact [1]. Production of a small ‘immunity’ protein called CdiI protects CDI^+^ bacteria from autoinhibition. CDI systems were subsequently found to be widespread amongst proteobacteria and to be polymorphic [2]. While the N-terminal ∼2800 amino acids of CdiA proteins (CdiA-NT) are highly similar, the C-terminal ∼350 amino acids (CdiA-CT) are highly variable [2]. CdiI proteins also vary and they do so in an allele-specific manner [2]. The current model for CDI states that CDI^+^ bacteria deliver their toxic CdiA-CTs into the cytoplasm of CDI^−^ bacteria upon cell-cell contact [3] and the CdiA-CTs, in most cases, catalyze the degradation of tRNA or DNA molecules [2], [4], leading to growth inhibition or death of the target cell. If present in the target cell, cognate (encoded by the same allele), but not heterologous (encoded by a different allele), CdiI proteins bind to CdiA-CTs, blocking their nuclease activity [2]. CDI (i.e., killing or growth inhibition of one bacterium by another) has so far only been demonstrated between CDI^+^ bacteria and CDI^−^ bacteria. Whether CDI occurs between bacteria producing different CDI systems, the biological relevance of CDI, and the biological relevance of CDI system polymorphism are unknown.

CDI systems fall into two major classes: “*E. coli*-type,” which are present in many genera of proteobacteria, and “*Burkholderia*-type,” which are restricted to *Burkholderia* species and a few closely related species of *Ralstonia* and *Cupriavidus* [2], [5], [6]. To distinguish the two major classes, genes encoding *Burkholderia*-type CDI systems were named *bcp* instead of *cdi* [6]. *Burkholderia*-type CDI systems differ from *E. coli*-type by the presence of an additional small ORF in the locus (*bcpO*), a different gene order (*bcpAIOB* instead of *cdiBAI*), and a different motif at the junction between the constant and variable regions of the large BcpA/CdiA exoproteins (Nx(E/Q)LYN instead of VENN) [2], [6]. *Burkholderia* species are Gram-negative bacterial soil saprophytes [7], [8] and include *B. pseudomallei*, an NIAID Category B priority pathogen and CDC Tier 1 select agent, and *B. thailandensis*, which rarely causes human disease [9]. *B. pseudomallei* infections, which can be fatal, are acquired only from the environment [10]–[12]. Although we previously identified three groups of CDI systems present in *B. pseudomallei* and *B. thailandensis* based on amino acid (aa) sequence similarity amongst the constant regions of BcpA proteins (BcpA-NT), aa sequence similarity amongst BcpB proteins, and the presence of a signal sequence in the predicted BcpO protein [6], further analysis indicates that alleles in groups 2 and 3 are too similar to justify separation into distinct groups, so they are all included in current group 2 (Fig. S1) [2], [5], [6].

We recently showed that in addition to mediating interbacterial competition, the CDI system-encoding genes of *B. thailandensis* E264 are required for biofilm formation [13], a phenomenon that typically requires cooperation amongst individual bacteria. Although biofilm formation by *B. thailandensis* required the activity of the BcpA protein, it was independent of BcpAIOB-mediated interbacterial killing [13], revealing a role for CDI system proteins in a process other than interbacterial competition.

Hallmark of both *E. coli*-type and *Burkholderia*-type CDI systems is the polymorphic nature of CdiA-CT/BcpA-CT and CdiI/BcpI proteins, which vary both within and between species. For all systems studied so far, CdiI/BcpI proteins protect against CDI in an allele-specific manner [2], [4]–[6]. These observations suggest the intriguing hypothesis that the variable ‘poison-antidote’ feature of CDI systems allows bacteria to distinguish ‘self’ from ‘non-self’ and to inhibit the growth of non-self organisms, i.e., that CDI functions generally as a form of kin recognition in the establishment of sociomicrobiological communities. We set out to test this hypothesis in the biologically relevant context of polymicrobial biofilms.

## Results

### Strain construction and predicted BcpA-CT activities

**Figure 1:**
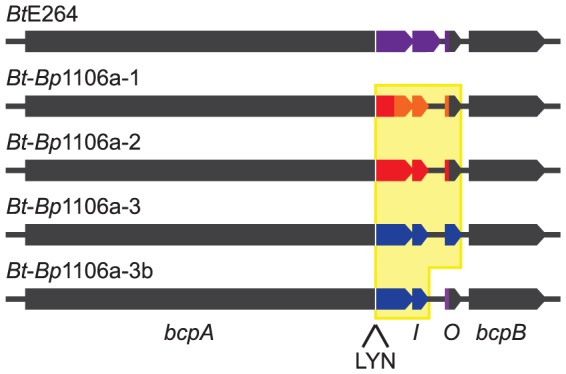
*Bt*E264 and *Bt*-*Bp* chimeric *bcpAIOB* loci diagram. Gray indicates *Bt*E264 DNA. Colors indicate variable DNA sequences encoding BcpA-CT, BcpI, and BcpO proteins from *Bt*E264 and *Bp*1106a. Yellow shading indicates *Bp*1106a DNA that replaced *Bt*E624 DNA.

*E. coli* and *B. thailandensis* strains producing chimeric CdiA or BcpA proteins with CdiA-CT or BcpA-CT domains encoded by different alleles have been shown to be capable of interbacterial competition, suggesting CdiA and BcpA proteins are modular, i.e., that the CdiA-CT and BcpA-CT domains function as independent interchangeable units [2], [4], [5]. However, in these experiments, the *cdiBAI* or *bcpAIOB* genes were expressed on multi-copy plasmids from inducible promoters, potentially obscuring the ability to discern subtle differences in CDI activity amongst the strains. We constructed four *B. thailandensis* E264 (*Bt*E264) derivatives by allelic exchange that contain chimeric *bcpAIOB* operons in the native site on the chromosome (chromosome I) and expressed from the native *Bt*E264 *bcpAIOB* promoter. We used *B. pseudomallei* strain 1106a (*Bp*1106a), which contains three different *bcpAIOB* alleles – each of which is present individually in other *B. pseudomallei* strains (Table 1) – as the source of DNA for these experiments. Two of the alleles from *Bp*1106a (alleles 1 and 2) are in the same group (group 1) as the *bcpAIOB* allele in *Bt*E264 and encode BcpO proteins that are predicted to differ from the *Bt*E264 BcpO protein only in their signal sequences. The third allele of *Bp*1106a (allele 3) is in group 2 and encodes a BcpO protein that is not predicted to contain a signal sequence (Fig. 1, Fig. S1). The *bcpO* gene of *Bt*E264 is required for proper function of BcpA during interbacterial competition [6].

**Table 1:** Distribution of *bcpAIOB* alleles.

*Bp*1106a *bcpAIOB* allele	Identical allele also found in:
*Bp*1106a-1	*Bp*BCC215-2
*Bp*1106a-2	*Bp*B7210-2, *Bp*1710b-1, *Bp*112-2, *Bp*Pasteur52237
*Bp*1106a-3	*Bp*E479, *Bp*DM98, *Bp*S13, *Bp*1710b-2, *Bp*1655, *Bp*B7210-1, *Bp*112-1

Anderson *et. al.* 2012.

Nikolakakis *et. al.* 2012.

In the first three chimeric strains constructed, DNA from the Nx(E/Q)LYN-encoding sequence that separates the conserved from the variable region of *bcpA* to the stop codon of *bcpO* from *Bt*E264 was replaced with the corresponding DNA from each *Bp*1106a *bcpAIOB* allele to generate strains *Bt-Bp*1106a-1, *Bt*-*Bp*1106a-2, and *Bt*-*Bp*1106a-3 (Fig. 1). The fourth strain, *Bt*-*Bp*1106a-3b, contains *Bp*1106a (*bcpAIOB* allele 3) DNA from the Nx(E/Q)LYN-encoding sequence of *bcpA* to the stop codon of *bcpI* (instead of *bcpO*) (Fig. 1). Therefore, strain *Bt*-*Bp*1106a-3b encodes the native *Bt*E624 BcpO predicted periplasmic lipoprotein, whereas strain *Bt*-*Bp*1106a-3 encodes the *Bp*1106a-3 BcpO protein that is predicted to be cytoplasmic. All strains grew equivalently in liquid culture (Fig. S2).

Although several CdiA-CTs and BcpA-CTs have been shown to function *in vitro* as nucleases [2], [4], [5], the activities of BcpA-CTs encoded by *Bt*E264, *Bp*1106a-1, and *Bp*1106a-2 are unknown. The C-terminus of BcpA-CT*_Bt_*_E264_ shares predicted secondary structure similarity to Holliday junction DNA resolvases and endonucleases from archeal species and substitution of aa predicted to be required for catalytic activity abrogate BcpA-mediated CDI and biofilm formation in *Bt*E264 [13], but the actual activity of BcpA-CT*_Bt_*_E264_ has not been determined. Attempts to identify functional domains within either BcpA-CT*_Bp_*_1106a-1_ or BcpA-CT*_Bp_*_1106a-2_ bioinformatically failed to yield a predicted activity. An allele identical to *Bp*1106a-3 (present in *B. pseudomallei* strain E479), however, has been shown to function as a tRNase [5].

### Chimeric BcpA proteins are capable of mediating CDI, but do so with decreased efficiency compared with the native protein

To measure CDI activity among strains that differ only in the specificity/activity of their BcpA and BcpI proteins, we used our chimeric strains in the colony biofilm interbacterial competition assay that we developed for use with *B. thailandensis* [6]. Inhibitor bacteria were mixed with target bacteria in a 1∶1 ratio (unless otherwise noted), deposited onto agar forming a spot approximately 9 mm in diameter, and incubated for 24 hours. Bacteria were picked from the center and edge of the formed colony biofilm, plated on agar containing appropriate antibiotics to distinguish the two strains, and the competitive indices (C.I.) were calculated. Using this assay, we showed previously that wild-type *Bt*E264 outcompeted a *Bt*Δ*bcpAIOB* strain by approximately 2.4 logs in the center and completely, i.e., only the wild-type inhibitor strain was recovered, along the edge of the colony biofilm by 24 hours (Table 2) [6]. The disparity between C.I. at the center and edge is presumably due to the fact that the force of pipetting pushes bacteria to the edge of the colony biofilm such that, at the beginning of the experiment, bacteria at the edge are all in contact with each other while those in the center do not contact other bacteria until they have replicated several times [6]. We can therefore measure CDI when different strains are in contact immediately and when they approach each other on a solid surface in the same assay.

**Table 2:** Summary of CDI results.

Inhibitor Strain	Target Strain	Center C.I. (Log)	Edge C.I. (Log)
* *Bt*E264	*Bt*Δ*bcpAIOB*	2.4	≥4.4
*Bt*-*Bp*1106a-1	*Bt*Δ*bcpAIOB*	1.4	≥4.6
*Bt*-*Bp*1106a-2	*Bt*Δ*bcpAIOB*	0.9	≥4.1
*Bt*-*Bp*1106a-3	*Bt*Δ*bcpAIOB*	0	0
*Bt*-*Bp*1106a-3b	*Bt*Δ*bcpAIOB*	0	0
* *Bt*E264Δ*bcpO*	*Bt*Δ*bcpAIOB*	1.2	1.7
*Bt*E264	*Bt*-*Bp*1106a-1	0.8	3.6
*Bt*E264	*Bt*-*Bp*1106a-2	0.8	2.3
*Bt*E264	*Bt*-*Bp*1106a-3	2.1	≥4.8
*Bt*E264	*Bt*-*Bp*1106a-3b	2.7	≥4.7
*Bt*-*Bp*1106a-1	*Bt*-*Bp*1106a-2	−0.2	−1.1

All competitions performed at 1∶1 inhibitor to target.

*Experiments performed in Anderson *et. al.* 2012.

**Figure 2:**
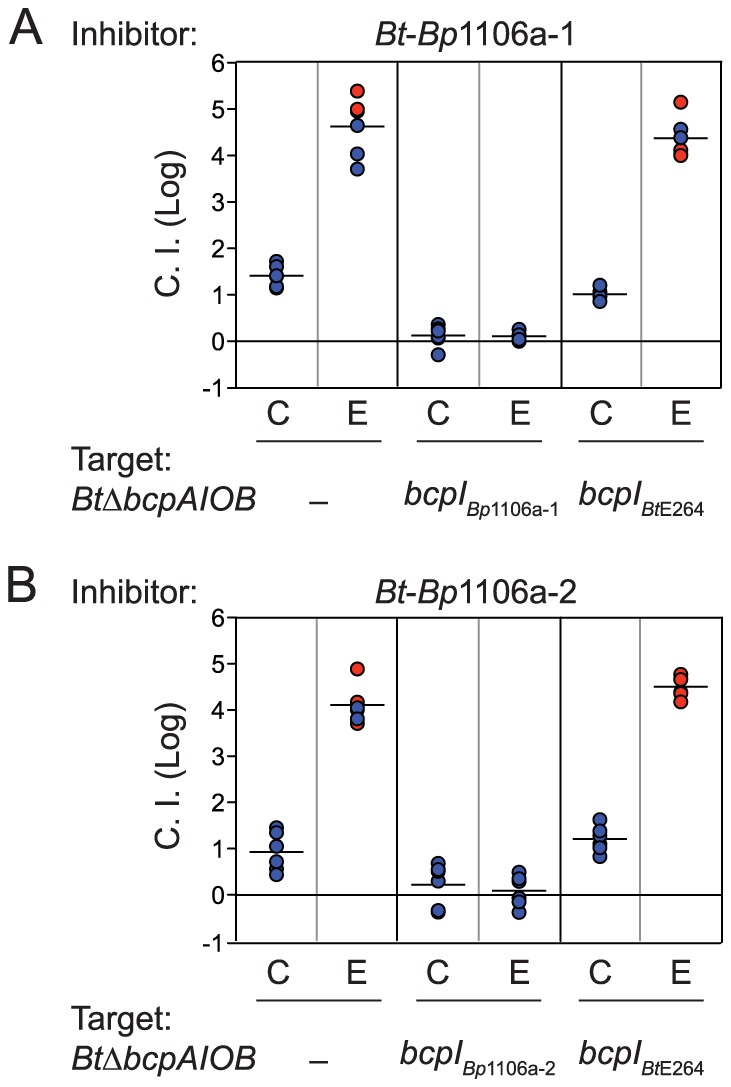
*Bt*-*Bp*1106a-1 and *Bt*-*Bp*1106a-2 mediated interbacterial competition. CDI-mediated competition between **A**) *Bt*-*Bp*1106a-1 or **B**) *Bt*-*Bp*1106a-2 (inhibitors) and *BtΔbcpAIOB* bacteria without immunity, with cognate immunity, or with heterologous immunity (targets). Samples of bacteria from the center (C) and edge (E) of colony biofilms taken after 24 hours were plated with antibiotics to determine the log competitive index (C.I. (Log)), blue data points. Red data points indicate only inhibitor bacteria were recovered, and the actual C.I. is therefore greater than or equal to the represented value.

*Bt*-*Bp*1106a-1 and *Bt*-*Bp*1106a-2 outcompeted *Bt*Δ*bcpAIOB* by approximately 1.4 logs and 0.9 logs in the center of the colony biofilm, respectively (Fig. 2). Along the edge of the colony biofilm, *Bt*-*Bp*1106a-1 and *Bt*-*Bp*1106a-2 outcompeted *Bt*Δ*bcpAIOB* by greater than or approximately equal to 4.6 logs and 4.1 logs, respectively, i.e., in some cases the chimeric strains completely outcompeted *Bt*Δ*bcpAIOB* bacteria and only inhibitor bacteria were recovered. Ectopic constitutive expression (from the *B. thailandensis rpsL* promoter, P_S12_) of cognate *bcpI* genes, but not of the heterologous *bcpI_Bt_*_E264_ gene, prevented *Bt*Δ*bcpAIOB* bacteria from growth inhibition by the chimeric strains, demonstrating that competition was indeed due to CDI and that the BcpI proteins tested here protect in an allele-specific manner (Fig. 2). These data indicate that these chimeric BcpA proteins are capable of mediating CDI. However, they do so to a lesser degree compared to the wild-type BcpA protein of *Bt*E264 (Table 2). These results suggest a hierarchy of potency with BpcA-CT*_Bt_*_E264_ being more toxic than BcpA-CT*_Bp_*_1106a-1_ or BcpA-CT*_Bp_*_1106a-2_. An alternative and equally plausible explanation is that the decreased CDI efficiency of the chimeric strains is due to species-specificity of BcpAIOB proteins, i.e., that *bcpA* alleles present in *B. thailandensis* strains encode proteins that are more effectively translocated to the cell surface, are recognized by target bacteria from native bacteria more effectively, or are more effective at inhibiting the growth of *B. thailandensis* strains than *B. pseudomallei* strains and vice versa.

### Chimeric strains *Bt*-*Bp*1106a-3 and *Bt*-*Bp*1106a-3b are non-functional for CDI

**Figure 3:**
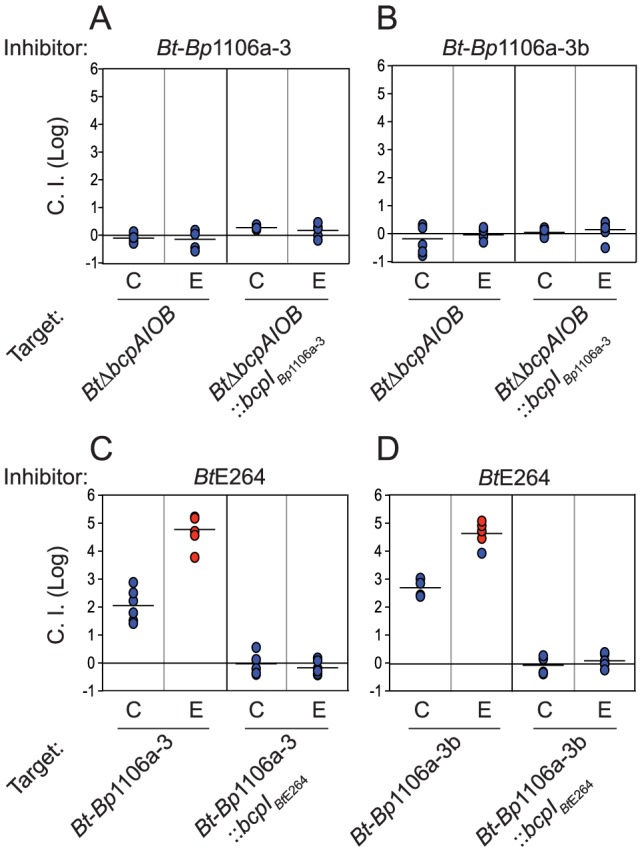
Lack of *Bt*-*Bp*1106a-3 and *Bt*-*Bp*1106a-3b mediated interbacterial competition. Samples of bacteria from the center (C) and edge (E) of colony biofilms taken after 24 hours were plated with antibiotics to determine the log competitive index (C.I. (Log)) (blue data points) for competitions between **A**) *Bt*-*Bp*1106a-3 or **B**) *Bt*-*Bp*1106a-3b (inhibitors) and *BtΔbcpAIOB* bacteria without immunity or with cognate immunity (targets), **C**) *Bt*E264 (inhibitor) and *Bt*-*Bp*1106a-3 without immunity or with cognate immunity (targets), **D**) *Bt*E264 (inhibitor) and *Bt*-*Bp*1106a-3b without immunity or with cognate immunity (targets). Red data points indicate only inhibitor bacteria were recovered, and the actual C.I. is therefore greater than or equal to the represented value.

*Bt*-*Bp*1106a-3 was not capable of out-competing *Bt*Δ*bcpAIOB* (Fig. 3A, first two columns), and constitutive expression of *bcpI_Bp_*_1106a-3_ in *Bt*Δ*bcpAIOB* had no effect on the C.I. (Fig. 3A, second set of columns). The *Bp*1106a-3 *bcpAIOB* allele is in a different group as the *Bt*E264, *Bp*1106a-1, and *Bp*1106a-2 *bcpAIOB* alleles (Fig. S1), and *Bt*-*Bp*1106a-3 bacteria encode a predicted cytosolic BcpO protein, whereas wild-type *Bt*E264, *Bt*-*Bp*1106a-1, and *Bt*-*Bp*1106a-2 each encode predicted periplasmic BcpO lipoproteins that are nearly identical (except for their signal sequences). We hypothesized that while BcpA-CT and BcpI proteins function in an allele-specific manner, BcpO proteins may be specific for a portion of the conserved region of BcpA (i.e., the BcpA-NT) or for BcpB. We therefore constructed strain *Bt*-*Bp*1106a-3b (Fig. 1). However, *Bt*-*Bp*1106a-3b was also unable to out-compete *Bt*Δ*bcpAIOB* (Fig. 3B). Furthermore, both *Bt*-*Bp*1106a-3 and *Bt*-*Bp*1106a-3b were out-competed by wild-type *Bt*E264, but were rescued by constitutive expression of *bcpI_Bt_*_E264_ (Fig. 3C and 3D), as if they were CDI^−^ bacteria. These results suggest that, for the alleles tested here at least, *bcpAIOB* group distinctions correspond to specificity of BcpAIOB proteins and imply that modularity may not extend beyond the individual groups, supporting the hypothesis that BcpA-CT (and possibly CdiA-CT) do not function independently of the rest of the CDI system proteins (Fig. S1).

### Interbacterial competition between strains expressing different *bcpA* alleles

**Figure 4:**
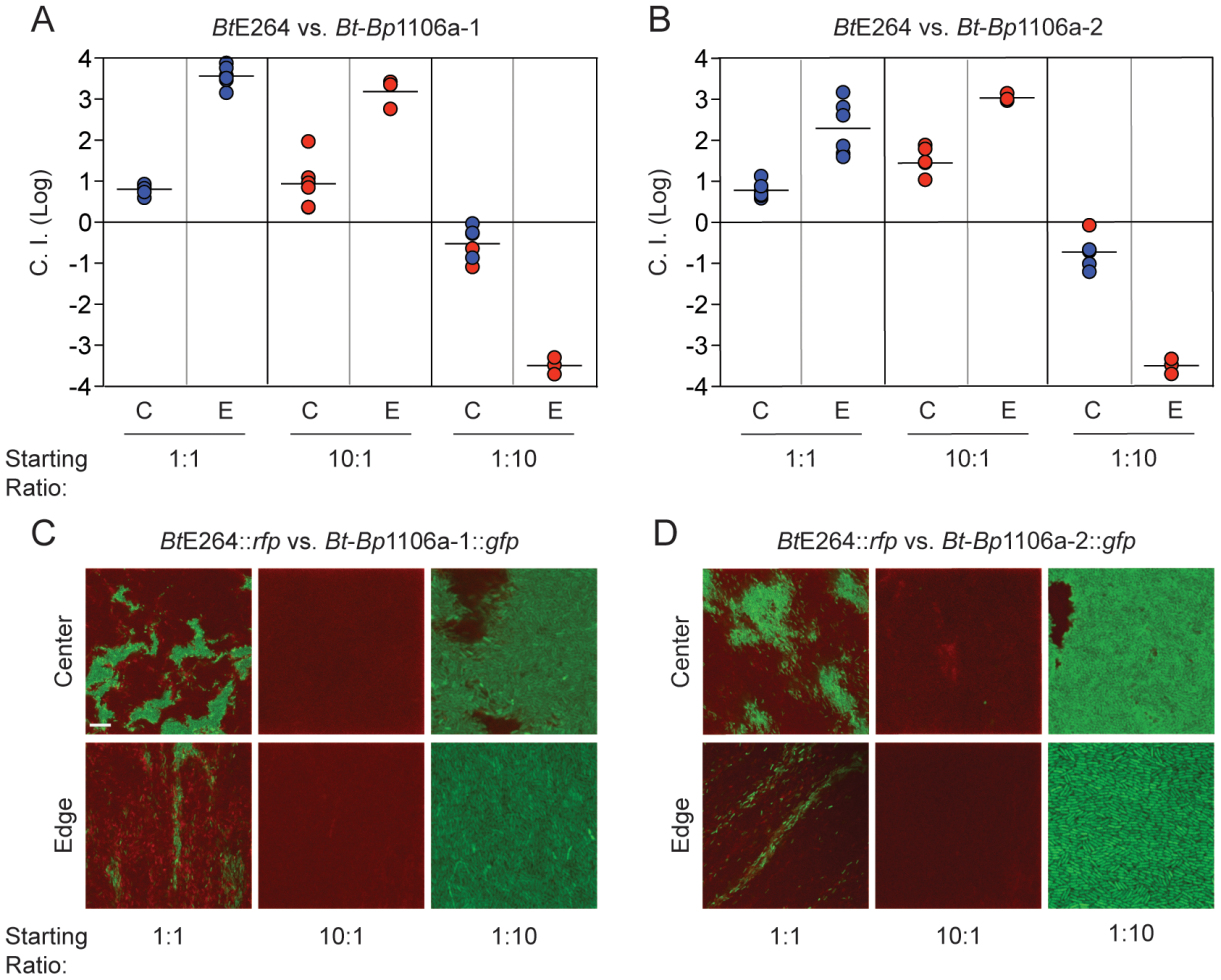
Competition between CDI^+^ bacteria expressing different *bcpA* alleles. The log competitive index (C.I. (Log)) is plotted for competitions between **A**) *Bt*E264 and *Bt*-*Bp*1106a-1 and **B**) *Bt*E264 and *Bt*-*Bp*1106a-2 at 24 hours in the center (C) and edge (E) of colony biofilms with starting ratios of 1∶1, 10∶1, and 1∶10. A positive C.I. (Log) indicates *Bt*E264 bacteria outcompeted the *Bt-Bp* chimeric strains, and negative C.I. (Log) indicates the *Bt-Bp* chimeric strains outcompeted *Bt*E264 bacteria. Blue data points indicate the C.I. (Log). Red data points indicate only the “winning” (outcompeting) strain was recovered, the actual C.I. is therefore greater than or equal to the represented value. **C**) and **D**) Microscopy of colony biofilms mixed with *Bt*E264::*rfp* and *Bt*-*Bp*1106a-1::*gfp* or *Bt*-*Bp*1106a-2::*gfp* at the indicated ratios. Images were taken of the center and edge at 24 hours. Note that bacteria completely span the field of view for all images taken (edge images were taken, therefore, just inside the edge). Scale bar = 10 µm.

To date, CDI-mediated interbacterial competition has only been demonstrated between bacteria possessing CDI systems and those that do not possess CDI systems, either naturally or due to genetic mutation. In their natural environment, however, it is likely that bacteria possessing different CDI systems will come into contact. To model inter-strain CDI, we conducted competition experiments with wild-type *Bt*E264, *Bt*-*Bp*1106a-1, and *Bt*-*Bp*1106a-2. When mixed at a ratio of 1∶1, wild-type *Bt*E264 (inhibitors) outcompeted *Bt*-*Bp*1106a-1 (targets) by 0.8 logs in the center and 3.6 logs along the edge of the colony biofilm (Fig. 4A, left columns). *Bt*E264 also outcompeted *Bt*-*Bp*1106a-2 (targets) by 0.8 logs in the center and 2.3 logs along the edge of the colony biofilm (Fig. 4B, left columns). The C.I.s from these competitions were less than those for the competition between wild-type *Bt*E264 and *Bt*Δ*bcpAIOB* (Table 2) [6], indicating that wild-type bacteria did not outcompete the chimeric strains as well as they outcompeted CDI^−^ bacteria.

In addition to measuring CDI by calculating the C.I., we visualized bacteria during competition in a colony biofilm by confocal microscopy. Wild-type *Bt*E264 expressing *rfp* from P_S12_ (*Bt*E264::*rfp*), were mixed at a 1∶1 ratio with *Bt*-*Bp*1106a-1 or *Bt*-*Bp*1106a-2 expressing *gfp* from P_S12_ (*Bt*-*Bp*1106a-1::*gfp* or *Bt*-*Bp*1106a-2::*gfp*, respectively) and spotted on agar. After 24 hours, *Bt*E264::*rfp* bacteria occupied a greater area of space in the center of the colony biofilm compared to either *Bt*-*Bp*1106a-1::*gfp* or *Bt*-*Bp*1106a-2::*gfp*, which were localized to independent patches of, most likely, clonal subpopulations surrounded by *Bt*E264::*rfp* (Fig. 4C and 4D, top left panels). Along the edge of the colony biofilm, *Bt*E264::*rfp* dominated the population (Fig. 4C and 4D, bottom left panels). However, *Bt*-*Bp*1106a-1::*gfp* and *Bt*-*Bp*1106a-2::*gfp* were present in what appeared as “thin streaks,” only a few cells wide [6].

When mixed at a wild-type to chimeric strain ratio of 10∶1, wild-type bacteria completely outcompeted the chimeric strains in both the center and the edge of the colony biofilm, indicated by only red data points (Fig. 4A and 4B, middle columns) and only *Bt*E264::*rfp* was observed by microscopy (Fig. 4C and 4D, middle panels). However, if *Bt*-*Bp*1106a-1 or *Bt*-*Bp*1106a-2 were present in 10–fold greater numbers than *Bt*E264 at the start of the assay, the chimeric strains outcompeted wild-type bacteria by almost 1 log in the center (which corresponds to a final ratio of 1∶100, wild-type to chimeric strain (Fig. 4A and 4B, right columns, 4C and 4D, top right panels)) and completely along the edge, indicated by the red data points (Fig. 4A and 4B, right columns) and only *Bt*-*Bp*1106a-1::*gfp* and *Bt*-*Bp*1106a-2::*gfp* were observed by microscopy (Fig. 4C and 4D, bottom right panels).

These data suggest that during co-culture on solid medium, both strains participate in CDI, i.e., each strain attempts to inhibit the other (and possibly themselves), presumably by delivering toxic BcpA-CT into the cytoplasm of neighboring bacteria (however, autoinhibition does not occur due to the presence of the cognate immunity BcpI protein in ‘self’ bacteria). The C.I.s calculated here, therefore, represent the net result of CDI activity between different bacteria. Furthermore, regardless of the reason for the decreased CDI efficiency of the chimeric strains (be it due to genetic manipulation or inherently weaker BcpA-CT activity), these data indicate that increased bacterial numbers can compensate for decreased efficiency.

### Relative population sizes influence competition outcome

**Figure 5:**
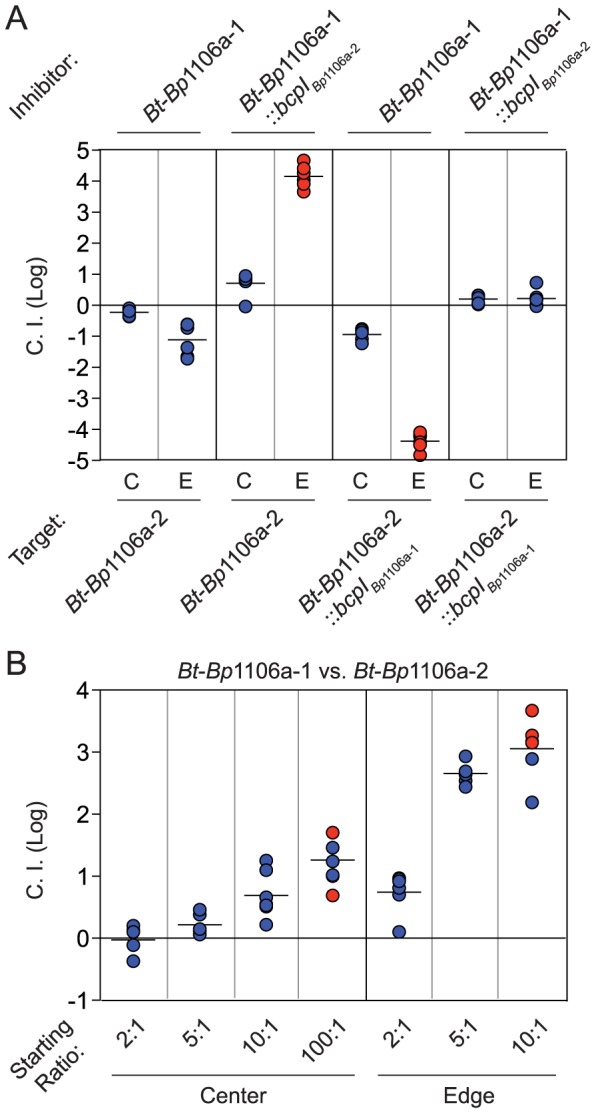
Dependence of bacterial numbers on CDI-mediated competition between opposing CDI^+^ bacteria. **A**) The log competitive index (C.I. (Log)) is plotted for competitions between *Bt*-*Bp*1106a-1 and *Bt*-*Bp*1106a-2 bacteria at a 1∶1 starting ratio (with and without immunity as indicated) in the center (C) and edge (E) of colony biofilms. A positive C.I. (Log) indicates the inhibitor bacteria outcompeted the target bacteria, and a negative C.I. (Log) indicates the target bacteria outcompeted the inhibitor bacteria. **B**) The log competitive index (C.I. (Log)) is plotted for competitions between *Bt*-*Bp*1106a-1 and *Bt*-*Bp*1106a-2 bacteria at the indicated starting ratios. A positive C.I. (Log) indicates *Bt*-*Bp*1106a-1 bacteria outcompeted *Bt*-*Bp*1106a-2 bacteria. All samples were taken at 24 hours. Blue data points indicate the C.I. (Log). Red data points indicate only the “winning” (outcompeting) strain was recovered, the actual C.I. is therefore greater than or equal to the represented value.

To determine the effect of bacterial numbers on competition between strains with similar CDI efficiency, we competed the chimeric strains against each other. When competed at a 1∶1 ratio, *Bt*-*Bp*1106a-1 (designated as ‘inhibitors’ for the purpose of calculating the C.I.) was outcompeted by *Bt*-*Bp*1106a-2 (designated ‘targets’) only at the edge of the colony biofilm and with a low C.I. (1.1 logs) (Fig. 5A, first set of columns, Table 2). *Bt*-*Bp*1106a-1 constitutively expressing *bcpI_Bp_*_1106a-2_, however, outcompeted *Bt*-*Bp*1106a-2 in the center of the colony biofilm by 0.7 logs and completely along the edge (Fig. 5A, second set of columns). Reciprocally, *Bt*-*Bp*1106a-2 constitutively expressing *bcpI_Bp_*_1106a-1_ outcompeted *Bt*-*Bp*1106a-1 in the center by 0.9 logs and completely along the edge (Fig. 5A, third set of columns). When both chimeric strains constitutively expressed the *bcpI* allele corresponding to the other strain, no net competition was observed (Fig. 5A, fourth set of columns). These data further support the conclusion that both CDI^+^ strains in a colony biofilm actively participate in CDI, and that expression of immunity genes in target bacteria provides protection against CDI.

We next performed competitions between the chimeric strains at ratios of 2∶1, 5∶1, 10∶1, and 100∶1 (*Bt*-*Bp*1106a-1 to *Bt*-*Bp*1106a-2). In the center of the colony biofilms, the C.I.s increased steadily from 0 logs for a 2∶1 mixture, 0.2 logs for 5∶1, 0.7 logs for 10∶1, and ≥1.3 logs for 100∶1 (Fig. 5B). Along the edge however, there was a sharp increase in the C.I.s between competitions performed at a starting ratio of 2∶1 (0.7 logs) and 5∶1 (2.7 logs) (Fig. 5B). The competition was nearly complete (i.e., only *Bt*-*Bp*1106a-1 was recovered in most of the samples – red data points) with a starting ratio of 10∶1 (≥3.1 logs) (Fig. 5B) and was complete in every replicate at 100∶1 (data not shown). These data indicate that greater cell numbers favor a strain during CDI. However, the spatial arrangement (i.e., center vs. edge of a colony biofilm) of bacteria can strongly influence the benefits that a larger population will experience.

### *Bt* chimeric strains are defective for two distinct *bcpAIOB*-mediated functions early in biofilm formation

**Figure 6:**
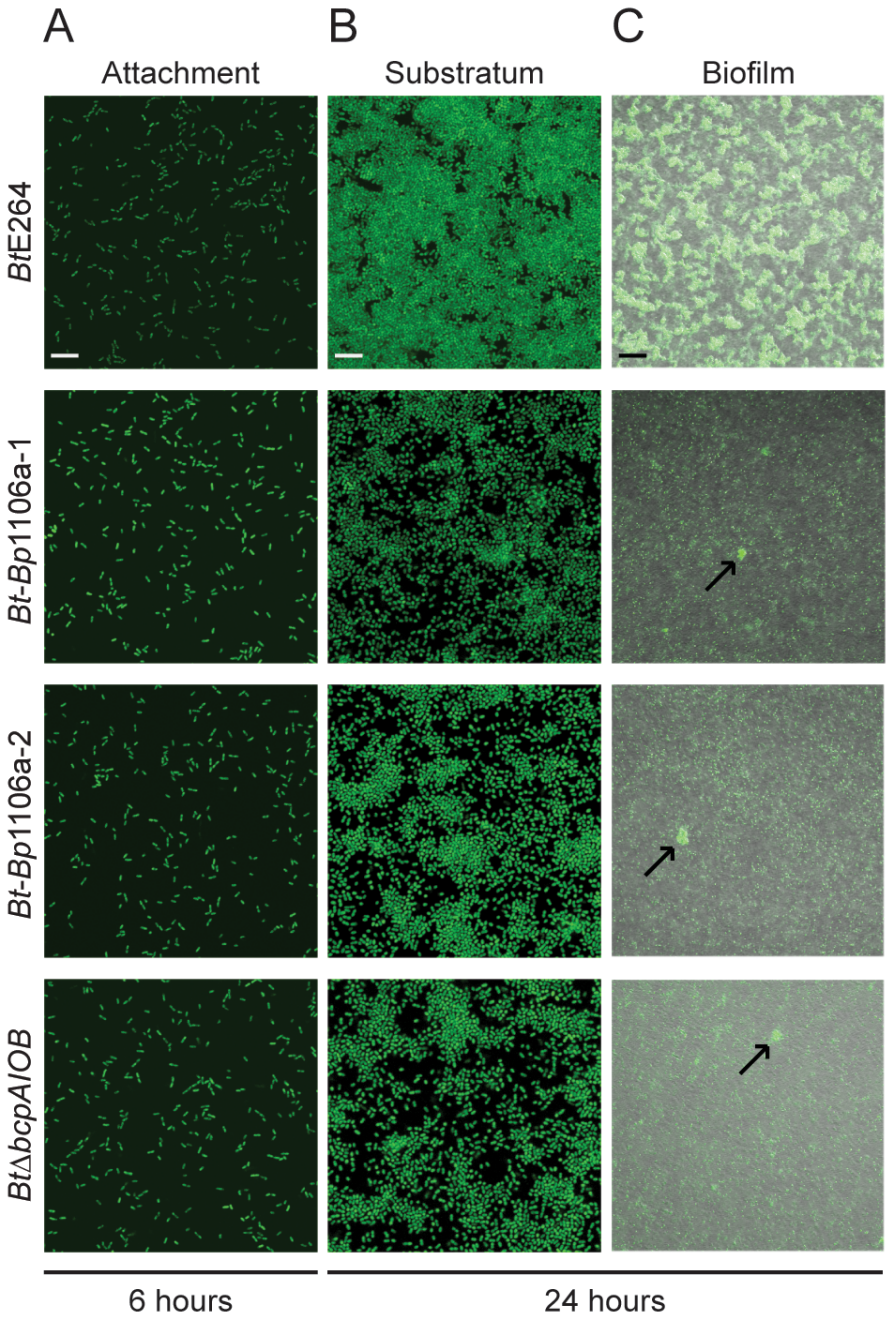
Analysis of *Bt-Bp* chimeric strains early in biofilm development. Confocal microscopy of early time points in biofilm development of *Bt*E264, *Bt*-*Bp*1106a-1, *Bt*-*Bp*1106a-2, and *Bt*Δ*bcpAIOB* bacteria, each constitutively expressing *gfp*. **A**) and **B**) Images taken at 6 and 24 hours in the plane of focus at the bottom of the biofilm (i.e., of bacteria directly attached to the glass bottom biofilm chambers). White scale bar = 10 µm. **C**) Fluorescent and DIC merged images taken at 24 hours in a plane of focus approximately half way between the top and bottom of the biofilm. Arrows indicate pillar structures. Black scale bar = 32 µm.

We next investigated the ability of chimeric strains *Bt*-*Bp*1106a-1 and *Bt*-*Bp*1106a-2 to form biofilms. Using an *in vitro* static biofilm assay [13], we showed previously that wild-type *Bt*E264 bacteria produce a biofilm in a BcpA activity-dependent, but interbacterial killing-independent, manner [13]. *Bt*-*Bp*1106a-1 and *Bt*-*Bp*1106a-2, along with *Bt*E264 and *Bt*Δ*bcpAIOB*, (expressing *gfp* constitutively) were each inoculated separately into glass bottom chambers and allowed to incubate statically at 37°C. At various time points, the biofilms were washed to remove any planktonic bacteria and imaged by confocal laser scanning microscopy (CLSM). After 6 hours, *Bt*E264, *Bt*-*Bp*1106a-1, *Bt*-*Bp*1106a-2, and *Bt*Δ*bcpAIOB* each attached to the glass substrate equivalently (Fig. 6A). After 24 hours however, *Bt*-*Bp*1106a-1, *Bt*-*Bp*1106a-2, and *Bt*Δ*bcpAIOB* each replicated to fill in the substratum considerably less than *Bt*E264 (Fig. 6B). Furthermore, low magnification images of biofilms formed by each strain at 24 hours revealed a dramatic defect of the chimeric strains (and *Bt*Δ*bcpAIOB*) in frequency of pillar structure formation (i.e., spatially confined and coordinated upward growth of bacteria) (Fig. 6C). (Important to note is that the images in Fig. 6C of *Bt*-*Bp*1106a-1, *Bt*-*Bp*1106a-2, and *Bt*Δ*bcpAIOB* biofilms were selected for the presence of pillar structures – not every field of view contained pillar structures, whereas every field of view of the *Bt*E264 biofilm looked similar to the image shown.) These data indicate that not only are the *bcpAIOB* genes important, at a minimum, for forming the substratum prior to biofilm development, but that the native *Bt*E264 *bcpA* allele is required for this phenotype. Additionally, these data suggest that the *bcpAIOB* genes are also important for initiating the formation of pillar structures.

**Figure 7:**
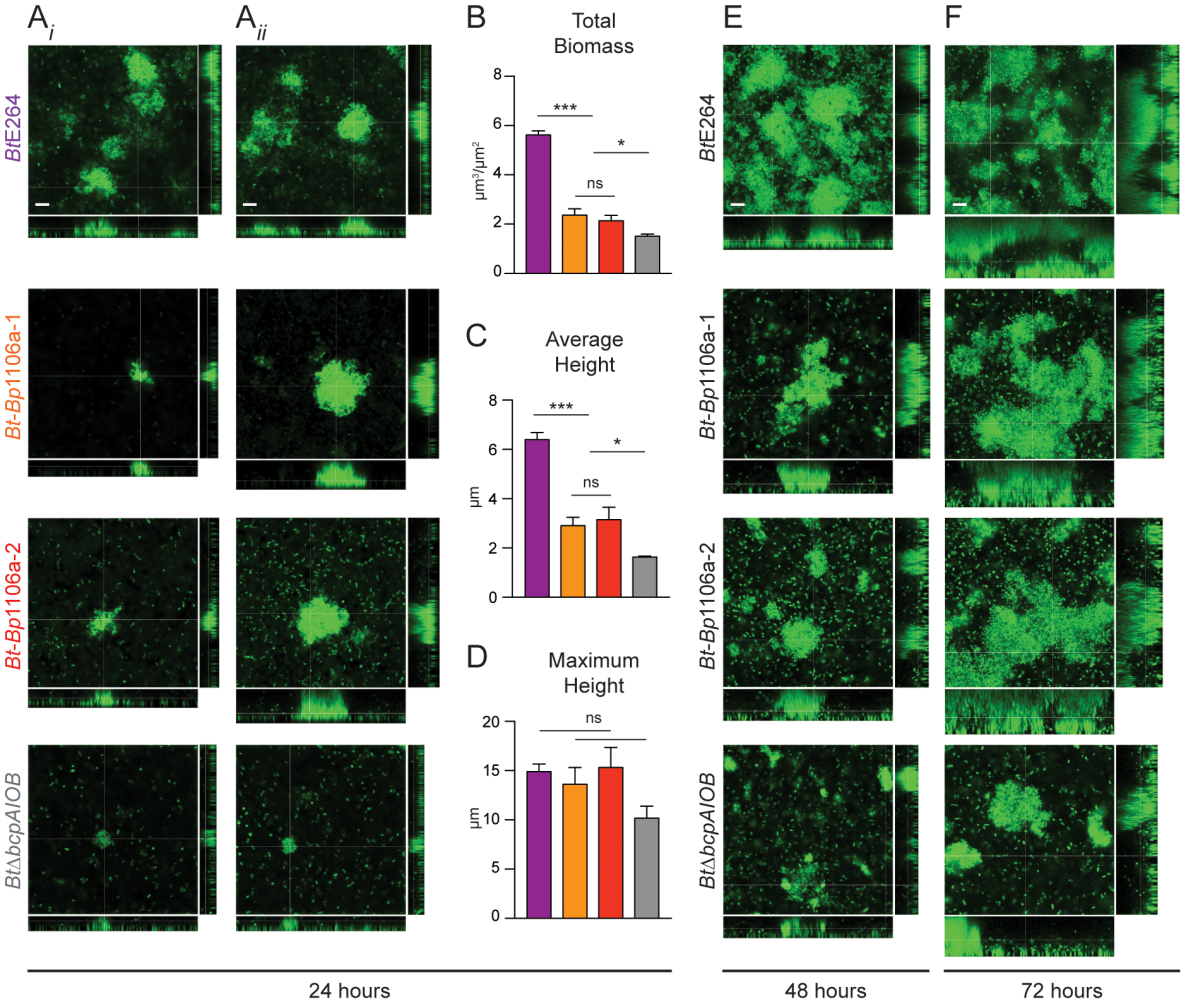
Analysis of pillar structure formation throughout biofilm development. CLSM and quantification of representative pillar structures in biofilms formed by *Bt*E264, *Bt*-*Bp*1106a-1, *Bt*-*Bp*1106a-2, and *Bt*Δ*bcpAIOB* bacteria, each expressing *gfp* constitutively. **A**) Images in A*i*) and A*ii*) represent examples of small and large, respectively, pillar structures formed by *Bt-Bp* chimeric strains, and represent typical pillar structures in both cases for *Bt*E264 and *Bt*Δ*bcpAIOB* bacteria, at 24 hours post inoculation. Z stack image cross-sections are shown in the plane parallel to the glass coverslip (shown by large center image) at 6 µm above the substratum. Scale bar = 10 µm. For each of the strains in A), COMSTAT analysis of Z stacks collected after 24 hours of biofilm development were used to determine **B**) total biomass, **C**) average height, and **D**) maximum height of the biofilms. *Bt*E264, purple bars; *Bt*-*Bp*1106a-1, orange bars; *Bt*-*Bp*1106a-2, red bars; and *Bt*Δ*bcpAIOB*, gray bars. Bars represent the mean of at least three independent experiments and error bars indicate the SEM. Significance was determined by two-tailed *t*-tests; * *p*<0.05, *** *p*<0.0001. **E**) and **F**) CLSM images of representative large pillar structures formed by the indicated strains at 48 and 72 hours, respectively, post inoculation. Z stack image cross-sections were taken at 6 µm (48 hours) or 10 µm (72 hours) above the substratum. Scale bar = 10 µm.

We located individual pillar structures under high magnification by searching multiple fields for the chimeric and *Bt*Δ*bcpAIOB* strains. (Again, pillar structures in biofilms formed by the wild-type strain were found in every field.) The chimeric strains mostly formed very small aggregates, resembling those of *Bt*Δ*bcpAIOB* (Fig. 7A*i*). However, in some rare cases the chimeric strains formed “large” pillars that resembled those of wild-type *Bt*E264 (Fig. 7A*ii*). Quantification of a representative compilation of pillar structures (both large and small) from multiple biofilms formed at 24 hours by each of the strains indicated that the total biomass and average height of the *Bt*-*Bp*1106a-1 and *Bt*-*Bp*1106a-2 biofilms were significantly less than those of wild-type *Bt*E264 biofilms; however, they were significantly more than those of *Bt*Δ*bcpAIOB* biofilms (Fig. 7B and 7C). The maximum height of biofilms formed, on the other hand, was not statistically different for wild-type *Bt*E264 and *Bt*-*Bp*1106a-1 and *Bt*-*Bp*1106a-2 bacteria; however, it was also not statistically different for *Bt*-*Bp*1106a-1 and *Bt*-*Bp*1106a-2 and *Bt*Δ*bcpAIOB* biofilms, likely due to the variability of pillars formed by the chimeric strains (Fig. 7D). (The maximum height of *Bt*E264 and *Bt*Δ*bcpAIOB* biofilms were statistically different *p*<0.0001, as previously demonstrated [13].)

We also examined biofilm formation after 48 and 72 hours. Again, when we specifically located large pillar structures by searching multiple fields, those formed by chimeric strains *Bt*-*Bp*1106a-1 and *Bt*-*Bp*1106a-2 appeared to be intermediate in biomass and height compared to those formed by *Bt*E264 and *Bt*Δ*bcpAIOB* (Fig. 7E and 7F). These data indicate that the chimeric strains are capable of forming biofilms with large pillar structures (unlike *Bt*Δ*bcpAIOB*); suggesting that the initiation of pillar structures is a unique function of BcpAIOB that is independent of substratum coverage.

**Figure 8:**
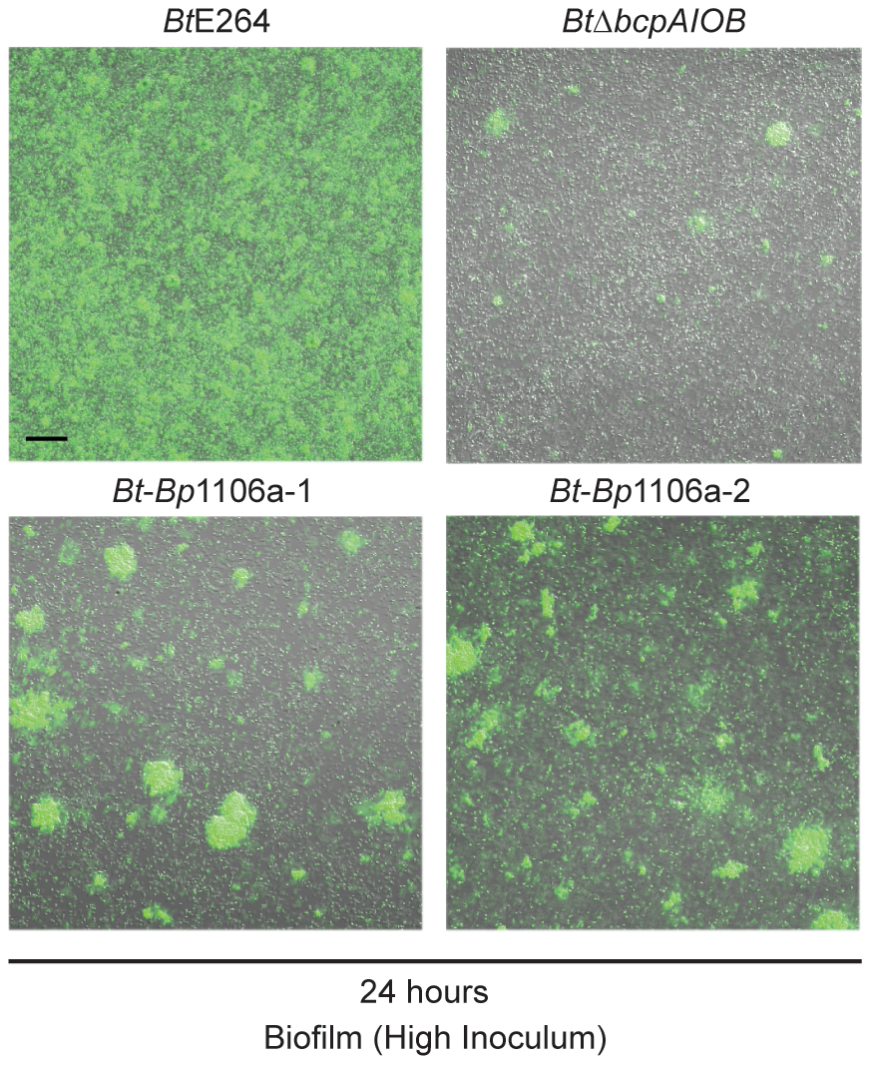
Pillar structure formation by strains at a higher inoculum. Confocal microscopy of biofilms formed with the “high inoculum”. Fluorescent and DIC merged images taken at 24 hours in a plane of focus approximately half way between the top and bottom of biofilms formed by *Bt*E264, *Bt*-*Bp*1106a-1, *Bt*-*Bp*1106a-2, and *Bt*Δ*bcpAIOB* bacteria, each constitutively expressing *gfp*. Black scale bar = 32 µm.

Because the chimeric strains (and *Bt*Δ*bcpAIOB*) formed a less dense substratum in the biofilm than wild-type bacteria (Fig. 6B), and also subsequently formed dramatically fewer pillar structures (Fig. 6C), we tested the hypothesis that a greater inoculum of the chimeric strains (as well as *Bt*Δ*bcpAIOB*) would lead to more pillar structures formed in the biofilm. We inoculated the biofilms with 10–fold greater numbers of bacteria (“high inoculum”) compared to the standard inoculum, and found that *Bt*-*Bp*1106a-1 and *Bt*-*Bp*1106a-2 did in fact form more pillar structures at 24 hours, which also appeared to be larger in size, compared to biofilms formed using the standard inoculum (Fig. 8). *Bt*Δ*bcpAIOB* also formed slightly more pillar structures at the high inoculum compared to the standard inoculum (Fig. 8). (Again note that the images in Fig. 8 of *Bt*-*Bp*1106a-1, *Bt*-*Bp*1106a-2, and *Bt*Δ*bcpAIOB* biofilms were selected for the presence of pillar structures – not every field of view contained pillar structures.) Wild-type bacteria, on the other hand, formed a thick, flat biofilm absent of individual pillar structures when the chambers were inoculated with this higher number of bacteria (Fig. 8). These data indicate that while increasing bacterial numbers enhanced pillar structure formation by the chimeric strains, it was not sufficient to pheno-copy *Bt*E264 biofilms.

Taken together, the data presented here are consistent with previously published data that indicate that the *bcpAIOB* genes play a role early in biofilm formation [13]. Our data suggest the *bcpAIOB* genes play two distinct roles: 1) formation of the substratum (for which the chimeric strains are equivalent to the *Bt*Δ*bcpAIOB* strain) and, 2) pillar structure initiation (for which the chimeric strains are defective in frequency compared to wild-type bacteria, but are significantly more capable than Δ*bcpAIOB* bacteria).

### BcpAIOB-mediated competition shapes biofilm community structure

**Figure 9:**
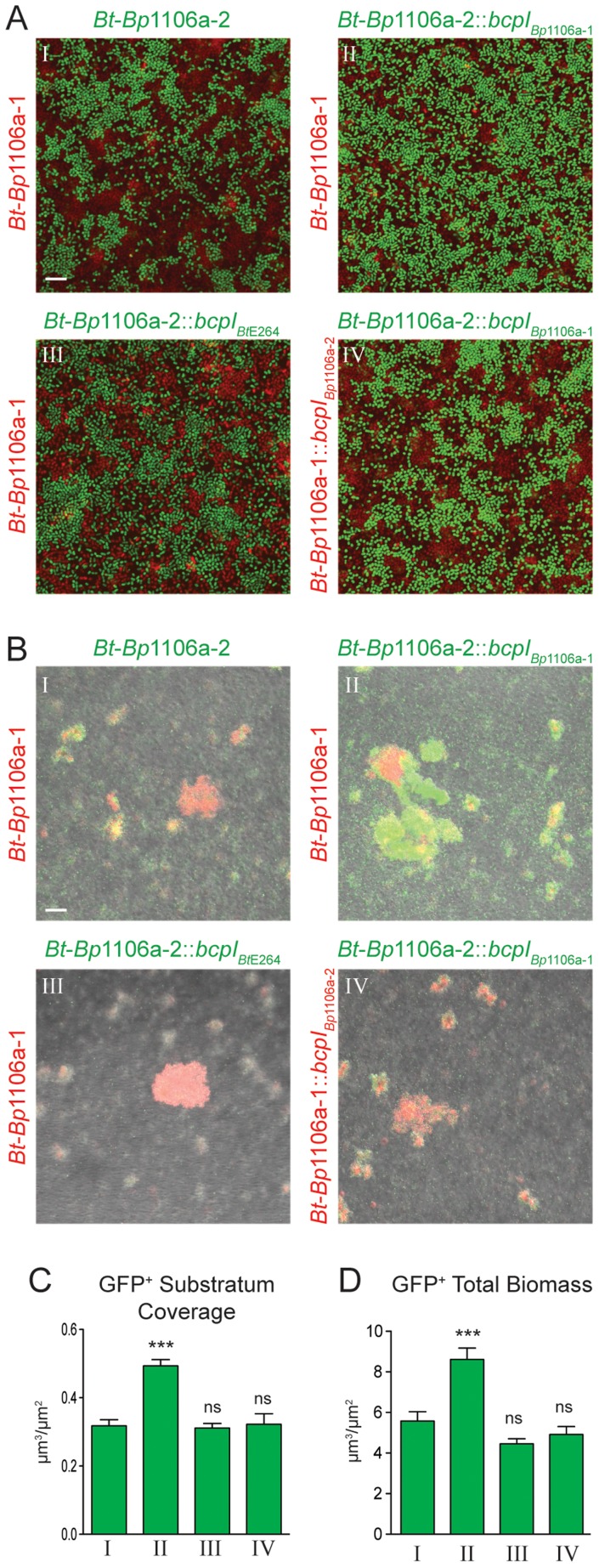
CDI system-mediated kind discrimination during polymicrobial biofilm formation of *Bt*-*Bp*1106a-1 and *Bt*-*Bp*1106a-2 bacteria. Confocal microscopy and quantitative analysis of polymicrobial biofilms 72∶1 starting ratio with the following strains: I. *Bt*-*Bp*1106a-1 and *Bt*-*Bp*1106a-2, II. *Bt*-*Bp*1106a-1 and *Bt*-*Bp*1106a-2::*bcpI_Bp_*_1106a-1_, III. *Bt*-*Bp*1106a-1 and *Bt*-*Bp*1106a-2::*bcpI_Bt_*_E264_, and IV. *Bt*-*Bp*1106a-1::*bcpI_Bp_*_1106a-2_ and *Bt*-*Bp*1106a-2::*bcpI_Bp_*_1106a-1_. For **A**) and **B**), strains in red carry a constitutive *rfp* gene, strains in green carry a constitutive *gfp* gene. For COMSTAT analysis in **C**) and **D**), green bars correspond to *gfp* expressing strain. Bars represent the mean of at least three independent experiments and error bars indicate the SEM. Significance was determined by two-tailed *t*-tests; *** *p*<0.0006; ns, not significant, comparing each strain to *Bt*-*Bp*1106a-2 (roman numeral I). **A**) Images taken of the plane at the bottom of the biofilm (substratum). Scale bar = 10 µm. **B**) Fluorescent and DIC merged images taken in a plane of focus approximately half way between the top and bottom of the biofilms. Scale bar = 32 µm. **C**) COMSTAT analysis of a single plane at the bottom of the biofilm (substratum), as shown in A). **D**) COMSTAT analysis of the total biofilm (i.e., all planes), from experiments shown in B).

To model polymicrobial biofilm formation, and to investigate the possibility that competition between strains producing different CDI system proteins occurs and alters biofilm structure, we co-inoculated chimeric strains *Bt*-*Bp*1106a-1 (constitutively expressing *rfp*) and *Bt-Bp*1106a-2 (constitutively expressing *gfp*) into glass bottom chambers at a 1∶1 ratio, allowed them to incubate statically for 72 hours, then washed the biofilms and imaged them by CLSM. We used the chimeric strains for these experiments because their ability to form mono-culture biofilms was similar. High magnification images were taken of the substratum (Fig. 9A) and low magnification images were taken of the total biofilm (Fig. 9B). We quantified the amount of GFP^+^ bacteria in the substratum (Fig. 9C) and in the total biofilm (Fig. 9D). Fields analyzed were selected blindly with regard to RFP and GFP. *Bt*-*Bp*1106a-1 (RFP^+^) and *Bt*-*Bp*1106a-2 (GFP^+^) were each present in the substratum at nearly equal amounts (Fig. 9AI). Curiously, the pillar structures formed were nearly always RFP^+^ bacteria (*Bt*-*Bp*1106a-1) (Fig. 9BI). We do not know why GFP^+^ pillars were only rarely found. However, the fact that pillars were composed of only one strain suggests that bacteria in pillar structures grow up clonally from the substratum.

Co-inoculation of biofilms with *Bt*-*Bp*1106a-1 (constitutively expressing *rfp*) and *Bt*-*Bp*1106a-2::*bcpI_Bp_*_1106a-1_ (constitutively expressing *gfp*), led to a significantly greater number of GFP^+^ bacteria in the substratum (Fig. 9AII, 9CII), indicating that CDI occurs in the substratum and was responsible for keeping the number of the two strains relatively equal when neither expressed the *bcpI* gene corresponding to the other strain. Significantly more GFP^+^ pillar structures were also found in the biofilm (along with independent RFP^+^ structures) compared to the biofilm shown in BI (Fig. 9BII, 9DII).

We also co-inoculated biofilms with *Bt*-*Bp*1106a-1 (constitutively expressing *rfp*) and *Bt*-*Bp*1106a-2::*bcpI_Bt_*_E264_ (constitutively expressing *gfp*). Expression of a heterologous immunity gene in *Bt*-*Bp*1106a-2 had no effect on either strain's ability to adhere to and multiply in the substratum, both were present in equal numbers (Fig. 9AIII, 9CIII). While both RFP^+^ and GFP^+^ pillar structures were found in the biofilm, a greater proportion were RFP^+^ *Bt*-*Bp*1106a-1 (total GFP^+^ bacteria were not present in significantly greater amounts than in biofilms shown in BI) (Fig. 9BIII, 9DIII).

Finally, biofilms were co-inoculated with *Bt*-*Bp*1106a-1::*bcpI_Bp_*_1106a-2_ (constitutively expressing *rfp*) and *Bt*-*Bp*1106a-2::*bcpI_Bp_*_1106a-1_ (constitutively expressing *gfp*). Both strains were present in equal numbers in the substratum (Fig. 9AIV, 9CIV). The pillar structures formed in these biofilms, however, were mostly mixed with both RFP^+^ and GFP^+^ bacteria (Fig. 9BIV), suggesting that immunity to CDI-mediated competition allowed the strains to form and co-inhabit the same pillar structure.

Collectively these data indicate that CDI-mediated competition occurs during biofilm development in the substratum. This competition ultimately shapes the biofilm composition by influencing which strains participate in pillar structure formation. The data suggest that bacteria use CDI as a mechanism to form pillar structures composed of only ‘self’ bacteria (Fig. 9BI). Furthermore, if all bacteria are immune to CDI by other bacteria present (by expression of cognate immunity genes), then all bacteria are recognized as self, and pillar structures are composed of a mixed population of bacteria (Fig. 9BIV).

### CDI systems mediate competitive exclusion in biofilm communities

**Figure 10:**
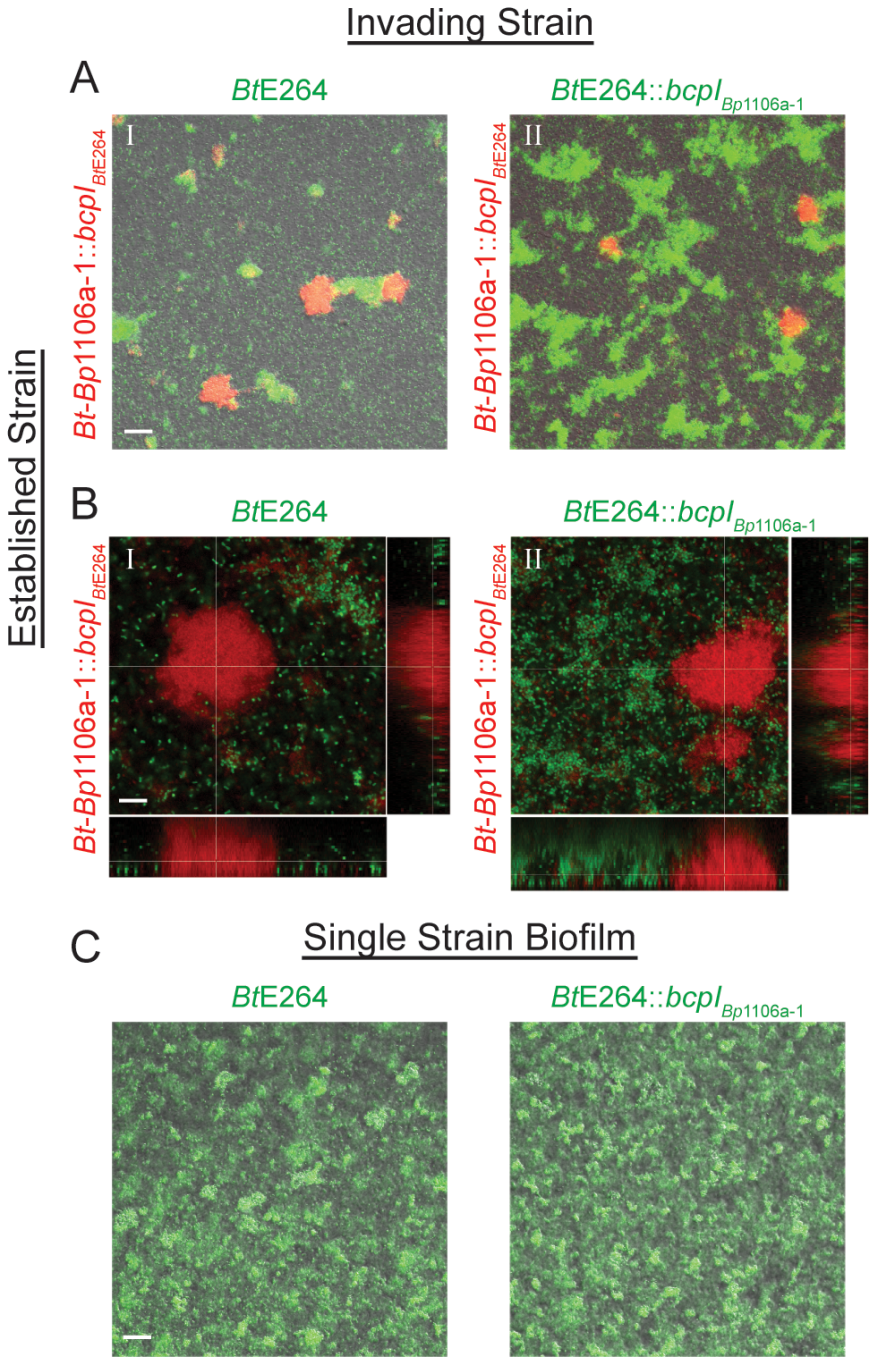
*bcpAIOB* allele-dependent competitive exclusion from an established community. **A**) Biofilms were inoculated with the “established strain,” *Bt*-*Bp*1106a-1::*bcpI_Bt_*_E264_, constitutively expressing *rfp*, incubated for 24 hours, washed, and then re-inoculated with the “invading strain,” I. *Bt*E264 or II. *Bt*E264::*bcpI_Bp_*_1106a-1_, constitutively expressing *gfp*, re-incubated for 48 hours, washed, and then imaged by confocal microscopy. Images shown are of fluorescent and DIC channels merged, taken in a plane of focus approximately half way between the top and bottom of the biofilms. Scale bar = 32 µm. **B**) CLSM of representative RPF^+^ pillar structures. Images are from the same experiment as in A). Z stack image cross-sections were taken at 10 µm above the substratum. Scale bar = 10 µm. **C**) Single strain biofilms formed by *Bt*E264 and *Bt*E264::*bcpI_Bp_*_1106a-1_, constitutively expressing *gfp*, 24 hours post inoculation. Images shown are of fluorescent and DIC channels merged, taken in a plane of focus approximately half way between the top and bottom of the biofilms. Scale bar = 32 µm.

To investigate whether CDI systems, as well as their diversity and allele-specificity, play a role in allowing bacteria to incorporate into pre-existing biofilms, we developed a “biofilm invasion assay.” We inoculated chambers with *Bt*-*Bp*1106a-1::*bcpI_Bt_*_E264_ (constitutively expressing *rfp*) and allowed them to establish a biofilm for 24 hours. (We provided the chimeric strain with the *Bt*E264 *bcpI* gene to protect the chimeric strain from CDI by the invading strain (*Bt*E264) because the chimeric strain is inherently less efficient at biofilm formation than *Bt*E264 (Figs. 6, 7, 8) and we sought to only test CDI by the established strain against the invading strain.) After 24 hours, the biofilm was washed and all non-adherent bacteria were removed. *Bt*E264 or *Bt*E264::*bcpI_Bp_*_1106a-1_ (each constitutively expressing *gpf*) (“invading strains”), were then introduced to the biofilm along with fresh medium, then incubated for an additional 48 hours. The biofilms were washed again and imaged by CLSM. Images were taken at low magnification to determine the number and distribution of large pillar structures (however, selection of fields was blind to the quantity of RFP^+^ and GFP^+^ structures) (Fig. 10A), and images were taken at high magnification and specifically focused on pillar structures of the established strain (RFP^+^ bacteria) to determine if the invading strain was able to incorporate into pre-existing structures (Fig. 10B).

RFP^+^ pillar structures composed of *Bt*-*Bp*1106a-1::*bcpI_Bt_*_E264_ were present in nearly all fields at low magnification for both experiments (Fig. 10AI, 10AII). For biofilms that were subsequently invaded with *Bt*E264, GFP^+^ pillar structures were also present in nearly all fields (Fig. 10AI). Strikingly however, when the biofilm established by *Bt*-*Bp*1106a-1::*bcpI_Bt_*_E264_ was invaded with *Bt*E264::*bcpI_Bp_*_1106a-1_ (a strain expressing the cognate immunity gene to the established strain), dramatically more GFP^+^ pillar structures formed (Fig. 10AII). (*Bt*E264 and *Bt*E264::*bcpI_Bp_*_1106a-1_ each formed equivalent biofilms in mono-culture after 24 hours (Fig. 10C)). These data indicate that expression of *bcpI_Bp_*_1106a-1_ was critical for *Bt*E264 to participate in the *Bt*-*Bp*1106a-1::*bcpI_Bt_*_E264_ biofilm. Collectively, these data strongly suggest that the BcpAIOB-mediated competition that occurs during biofilm development functions as a mechanism for competitive exclusion to inhibit non-self bacteria from incorporating into pre-established niches.

Upon examination of RFP^+^ pillar structures at high magnification for both experiments, we found no GFP^+^ bacteria co-existing in these structures (Fig. 10B). GFP^+^ pillar structures were found more frequently in biofilms in which the invading strain carried the cognate *bcpI* immunity gene to the established strain (Fig. 10BII), however these were independent structures. These data suggest that there is a critical point during development (likely prior to 24 hours) when planktonic bacteria in the medium (or bacteria localized nearby in the substratum) are unable to incorporate into growing pillar structures, regardless of their distinction as self or non-self.

## Discussion

Although the polymorphic nature of CDI systems and the fact that immunity proteins protect against CDI in an allele-specific manner suggest that bacteria may use CDI systems to recognize self and eliminate non-self bacteria from their immediate surroundings, this hypothesis has not been tested in a biologically relevant context. Our goal was to understand the significance of CDI system diversity in *Burkholderia* species. Specifically, we wanted to investigate the consequence of competition between strains producing different CDI systems with the ultimate goal of understanding how CDI amongst different strains and/or species affects bacterial communities in their natural environment. However, members of the *Burkholderia* genus are extremely heterogeneous, even within single species [14], [15], and therefore many variables exist even when comparing the most closely related strains in a controlled laboratory environment. To alleviate the complication of strain heterogeneity, we took a reductionist approach and constructed strains that differed only in the BcpA-CT and BcpI proteins they produced and used them to model inter-strain competition.

The chimeric strains *Bt*-*Bp*1106a-1 and *Bt*-*Bp*1106a-2 were able to mediate CDI against *Bt*Δ*bcpAIOB* in our interbacterial competition assay. However, they did so with less efficiency than wild-type *B. thailandensis* (Table 1). It has been hypothesized that a hierarchy of potency may exist among the toxic BcpA-CT/CdiA-CT encoded by *bcpAIOB* and *cdiBAI* alleles [5], [6]. The decreased CDI efficiency of *Bt*-*Bp*1106a-1 and *Bt*-*Bp*1106a-2 compared to wild-type *Bt*E264 could be due to differences in the intracellular toxicity of BcpA-CT*_Bp_*_1106a-1_ and BcpA-CT*_Bp_*_1106a-2_ compared with that of BcpA-CT*_Bt_*_E264_, supporting this hypothesis. Testing this hypothesis rigorously will require identifying and characterizing the catalytic activities and the substrates of each of the BcpA-CTs used in our analysis – the goals of future studies. It is also possible, however, that decreased CDI activity by the chimeric strains is due to strain and/or species specificity rather than (only) differences in enzymatic activity. For example, the true (or optimum) substrate for each BcpA-CT may be present only in the strain or species in which it is natively produced, and therefore BcpA-CT*_Bp_*_1106a-1_ and BcpA-CT*_Bp_*_1106a-2_ may be more toxic to *B. pseudomallei* than they are to *B. thailandensis*, and vice versa.

Alternatively, the chimeric BcpA proteins may be less efficient than the wild-type protein because BcpA proteins (and possibly CdiA proteins) are not truly modular, i.e., BcpA-CT (and CdiA-CT) may not function as independent interchangeable units. Although the ‘constant’ regions of BcpA*_Bt_*_E264_, BcpA*_Bp_*_1106a-1_, and BcpA*_Bp_*_1106a-2_ (i.e., BcpA-NT) are very similar, they are not identical (BcpA-NT*_Bt_*_E264_ and BcpA-NT*_Bp_*_1106a-1_ are 94% similar and 91% identical, and BcpA-NT*_Bt_*_E264_ and BcpA-NT*_Bp_*_1106a-2_ are 87% similar and 80% identical). It is possible that allele-specific interactions between different domains of BcpA or between BcpA and BcpB are required for proper BcpA secretion and translocation to the bacterial cell surface, BcpA recognition and binding of a receptor protein on target bacteria, and/or delivery of BcpA-CT into target cells and these differences could account for the different CDI activities of the chimeric strains.

Lack of modularity was clearly evident from our results with chimeric strains *Bt*-*Bp*1106a-3 and *Bt*-*Bp*1106a-3b, which were not capable of mediating CDI at all. Deficiency in CDI activity was likely not due to inactivity of BcpA-CT*_Bp_*_1106a-3_, as this domain has been characterized to have tRNase activity in *E. coli* [5]. Furthermore, a chimeric *bcpAIOB* locus that encoded the BcpA-CT and BcpI proteins from an allele identical to *Bp*1106a-3 was shown to be capable of interbacterial competition in *B. thailandensis* when fused to BcpA-NT from *Bp*1026b [5], indicating that this BcpA-CT can inhibit *B. thailandensis* when fused to a different BcpA-NT. In this case, however, the functional chimera resulted from swapping domains between alleles within group 2 [5] (Fig. S1), which are highly similar. Our *Bt*-*Bp*1106a-3 and *Bt*-*Bp*1106a-3b chimeras resulted from swapping domains between alleles in groups 1 and 2 (Fig. S1), which are less similar. Alleles in group 1 and group 2 differ in aa sequence of their BcpA-NT, BcpB, and BcpO proteins. BcpO proteins from group 1 are predicted periplasmic lipoproteins whereas BcpO proteins from group 2 are predicted cytoplasmic proteins. Although we hypothesized that differences in BcpO*_Bt_*_E264_ and BcpO*_Bp_*_1106a-3_ were responsible for the inactivity of chimeric strain *Bt*-*Bp*1106a-3, that was not the case as chimeric strain *Bt*-*Bp*1106a-3b was also inactive. Together, these data suggest that interactions occur between the BcpA-CT and BcpA-NT and/or BcpB, and that these interactions are required for proper function of BcpAIOB proteins during CDI. Our future studies will be aimed at identifying the regions within BcpAIOB proteins that dictate allele-specificity and modularity of these systems.

Despite their decreased interbacterial competition efficiency compared to wild-type *Bt*E264, chimeric strains *Bt*-*Bp*1106a-1 and *Bt*-*Bp*1106a-2 were useful for studying the role of diversity among CDI systems. Prior to our study, the consequence of competition between CDI^+^ bacteria producing different toxin and immunity proteins had not been investigated. Our data indicate that when bacteria expressing different *bcpAIOB* alleles interact, both engage in CDI and the outcome of the competition is influenced by both the potency/efficiency of each CDI system and relative bacterial cell numbers at the start of competition. When present in equal numbers, the ‘stronger’ strain wins the competition, but, if given a 5 to 10-fold cell number advantage, even a ‘weak’ strain can eliminate a much stronger competitor. These data imply that even if a hierarchy of *bcpAIOB* allele potency exists amongst wild-type bacteria in nature, the advantage conferred by greater potency may be limited to situations in which the bacteria are present in relatively equal numbers. In addition to highlighting the complexity of CDI systems and the manner in which they function, these results underscore the importance of interpreting data carefully and qualifying conclusions appropriately based on the experimental design.

Although they were more proficient at biofilm formation than the *Bt*Δ*bcpAIOB* mutant, the chimeric strains were also defective at both biofilm substratum coverage and pillar initiation compared with wild-type *B. thailandensis*. Since we do not yet understand the mechanism by which BcpA contributes to biofilm development, it is difficult to speculate why the chimeric strains are defective for this process. As with competition, perhaps the chimeric proteins experience suboptimal secretion or translocation to the bacterial cell surface or altered interaction with other surface proteins. Perhaps the substrate that BcpA-CT acts upon to mediate biofilm formation is species-specific or perhaps due to differences in enzymatic activity or other function, only a subset of BcpA/CdiA proteins overall mediates biofilm formation and the *Bp*1106a-1 and *Bp*1106a-2 systems do not fall into this group. We are currently investigating the mechanistic bases for why the chimeric strains are less efficient than wild-type *B. thailandensis* at CDI and biofilm formation, including determining the secretion, topology, and activity of the wild-type and chimeric BcpA proteins on the bacterial surface.

As with CDI, although the chimeric strains did not form biofilms efficiently and despite the fact that the underlying mechanistic basis for this inefficiency is not understood at this time, the chimeric strains were useful for examining the impact of CDI diversity on polymicrobial biofilm formation. The mechanisms used by bacteria to compete and cooperate with one another in diverse microbial communities in the environment are only beginning to be understood. Recent theory [16], as well as examples from *Bacillus subtilis* [17], [18] and *Enterococcus faecalis* [19], suggest that competition, rather than cooperation, is the driving force behind biofilm community development. While our previous work indicated that CDI-mediated competition occurs in the biofilm substratum but does not affect *B. thailandensis* biofilm formation in mono-culture [13], the data presented here indicate that CDI-mediated competition does ultimately affect the composition of polymicrobial biofilms (Fig. 9A), suggesting that bacteria use CDI as a mechanism to inhibit the incorporation of non-self bacteria into the community. Our current work (Fig. 10B), as well as our previous work [13], suggests that, like the model organism *B. subtilis* [20], *B. thailandensis* undergo a specific developmental process during biofilm formation which begins in the substratum and requires that bacteria grow upward into pillar structures, and therefore, planktonic bacteria do not get incorporated into pillar structures. We hypothesize that competition between bacteria in the substratum results in micro-communities of identical bacteria that then initiate pillar structure formation, resulting in pillars that are composed only of identical (with regard to CDI, at least) bacteria. Our data further support this idea because chimeric strains *Bt*-*Bp*1106a-1::*bcpI_Bp_*_1106a-2_ and *Bt*-*Bp*1106a-2::*bcpI_Bp_*_1106a-1_, each expressing the other strain's cognate immunity gene, formed mixed pillar structures in the biofilm when co-inoculated (Fig. 9B). This result is somewhat contradictory to our previous results in which *Bt*E264::*rfp* and *Bt*E264::*gfp* co-inoculated biofilms contained pillar structures primarily composed of either RFP^+^ or GFP^+^ pillars with a minor population of mixed structures [13]. This difference may be due to differences in biofilm formation efficiency between the chimeric and wild-type strains.

Our data suggest that CDI systems have evolved as a mechanism to allow microbes to discern those in the population that do not contribute to the persistence of their own genetic material, i.e., self versus non-self recognition [21]. Bacteria expressing different *bcpAIOB* alleles use the variability of BcpA-CT toxins as a means to discriminate kind amongst neighboring bacteria. Bacteria that are of the same kind (i.e., contain the same *bcpAIOB* allele), and are likely kin, will also produce the appropriate BcpI immunity protein, which allows the two strains to co-exist. However, lack of the appropriate immunity protein leaves bacteria susceptible to CDI-mediated competition, which can ultimately lead to exclusion from an entire pre-established community (Fig. 10), a likely scenario in nature. The situation is complicated by the incredible diversity in toxin-immunity pairs that exists even within a single bacterial species (for example, amongst *B. pseudomallei* strains) and the fact that bacteria may contain more than one CDI system-encoding locus in their genome. To complicate matters further, CDI systems are just one source of competitive diversity. Recent reports have shown that Type VI Secretion System-mediated interbacterial interactions also result in both inter- [22], [23] and intra-species [24], [25] bacterial competition that can potentially shape microbial communities. While we have provided evidence for a role for diversity among CDI systems, a far more challenging question of how diversity is generated in the first place still remains.

## Methods

### Culture conditions

*Burkholderia thailandensis* E264 is an environmental isolate [26]. Plasmids were maintained in *E. coli* DH5α and DH5αλpir and mated into *B. thailandensis* using the donor *E. coli* strain RHO3 [27]. *B. thailandensis* was cultured in low salt Luria-Bertani medium (LSLB, 0.5% NaCl) or M63 minimal medium (supplemented with 1 mM MgSO_4_, 0.2% glucose, and 0.4% glycerol) [28] supplemented, as appropriate, with 35 µg/ml chloramphenicol, 250 µg/ml kanamycin, 20 µg/ml tetracycline. *E. coli* strains were cultured in Luria-Bertani (LB) medium supplemented, as appropriate, with 100 µg/ml ampicillin, 35 µg/ml chloramphenicol, 50 µg/ml kanamycin, 20 µg/ml tetracycline, or 200 µg/ml diaminopamillic acid. Overnight cultures were aerated for ∼18 h at 37°C.

To measure the growth of the chimeric strains, triplicate overnight cultures were diluted to an OD600 = 0.04 in LSLB in a sterile 96-well polystyrene plate. The plate was incubated at 37°C with constant shaking and OD600 was measured every 15 min using an Infinite M200 Pro plate reader (Tecan).

### Strain construction

Chimeric strains *Bt*-*Bp*1106a-1, *Bt*-*Bp*1106a-2, *Bt*-*Bp*1106a-3, and *Bt*-*Bp*1106a-3b were constructed by allelic exchange. For each strain, three individual DNA fragments were PCR amplified: 1) 500 bp of *Bt*E264 DNA directly 5′ of the LYN-encoding region of *bcpA*, 2) *Bp*1106a DNA from the LYN-encoding region of *bcpA* to the stop codon of *bcpO* for *Bt*-*Bp*1106a-1, *Bt*-*Bp*1106a-2, and *Bt*-*Bp*1106a-3, or to the stop codon of *bcpI* for *Bt*-*Bp*1106a-3b, and 3) 500 bp of *Bt*E264 DNA directly 3′ to the stop codon of *bcpO* for *Bt*-*Bp*1106a-1, *Bt*-*Bp*1106a-2, and *Bt*-*Bp*1106a-3, or directly 3′ to the stop codon of *bcpI* for *Bt*-*Bp*1106a-3b. Fragments 1 and 2 were combined by overlap PCR. Fragment 3 was subsequently joined to the 3′ end of fragments 1+2 after restriction site digestion and ligation into allelic exchange vector pEXKm5 [27].

Strains expressing *bcpI* immunity genes and/or *gfp* or *rfp* genes were constructed using a mini-Tn7 system as described [29]. The pUC18miniTn7(TC) plasmid, conferring tetracycline resistance, was used to insert all P_S12_ *bcpI* constructs onto the chromosome of *Bt*E264 at either of two *att*Tn7 sites, as previously described [6]. *Bt*E264 was marked with constitutive *gfp* or *rfp* using the miniTn7-*kan*-*gfp* or miniTn7-*kan*-*rfp* plasmids, respectively [30], as previously described [6], [13]. Strains marked with antibiotic selection for interbacterial competition assays were obtained using either pUC18miniTn7(KM), conferring kanamycin resistance, or pUC18miniTn7(CM), conferring chloramphenicol resistance [6]. The *Bt*Δ*bcpAIOB*(KM^R^) and *Bt*Δ*bcpAIOB*(KM^R^)::*bcpI_Bt_*_E264_ strains were constructed as previously described [6]. The *Bt*E624::*gfp*(KM^S^) and *Bt*Δ*bcpAIOB*(KM^S^)::*gfp*(KM^R^) strains were constructed as previously described [13]. All strains and plasmids were verified by DNA sequencing (Eton BioScience). All relevant experimental strains are listed in Table 3.

**Table 3:** Relevant strain list.

Base Strain	*att*Tn7^1^ (Resistance)^2^	*att*Tn7^1^ (Resistance)^2^	Other	Experiment	Reference
*Bt*E264	–	–		Fig. 1	Brett *et. al.* 1998
*Bt*-*Bp*1106a-1	–	–		Fig. 1	This study
*Bt*-*Bp*1106a-2	–	–		Fig. 1	This study
*Bt*-*Bp*1106a-3	–	–		Fig. 1	This study
*Bt*-*Bp*1106a-3b	–	–		Fig. 1	This study
*Bt*E264	– (CM)	–		Fig. 3C, 3D, 4A, 4B	Anderson *et. al.* 2012
*Bt-Bp*1106a-1	– (CM)	–		Fig. 2A, 5A	This study
*Bt*-*Bp*1106a-2	– (CM)	–		Fig. 2B, 5A, 5B	This study
*Bt*-*Bp*1106a-3	– (CM)	–		Fig. 3A	This study
*Bt*-*Bp*1106a-3b	– (CM)	–		Fig. 3B	This study
*Bt*-*Bp*1106a-1	– (KM)	–		Fig. 4A, 5A, 5B	This study
*Bt*-*Bp*1106a-2	– (KM)	–		Fig. 4B	This study
*Bt*-*Bp*1106a-3	– (KM)	–		Fig. 3C	This study
*Bt*-*Bp*1106a-3b	– (KM)	–		Fig. 3D	This study
*Bt*Δ*bcpAIOB*	–	–	KM^R^	Fig. 2A, 2B, 3A, 3B	Anderson *et. al.* 2012
*Bt*Δ*bcpAIOB*	*bcpI_Bt_*_E264_ (TC)	–	KM^R^	Fig. 2A, 2B	Anderson *et. al.* 2012
*Bt*Δ*bcpAIOB*	*bcpI_Bp_*_1106a-1_ (TC)	–	KM^S^	Fig. 2A	This study
*Bt*Δ*bcpAIOB*	*bcpI_Bp_*_1106a-2_ (TC)	–	KM^S^	Fig. 2B	This study
*Bt*Δ*bcpAIOB*	*bcpI_Bp_*_1106a-3_ (TC)	–	KM^S^	Fig. 3A, 3B	This study
*Bt*-*Bp*1106a-3	*bcpI_Bt_*_E264_ (TC)	–		Fig. 3C	This study
*Bt*-*Bp*1106a-3b	*bcpI_Bt_*_E264_ (TC)	–		Fig. 3D	This study
*Bt*-*Bp*1106a-1	*bcpI_Bp_*_1106a-2_ (TC)	– (KM)		Fig. 5A	This study
*Bt*-*Bp*1106a-1	*bcpI_Bp_*_1106a-2_ (TC)	– (CM)		Fig. 5A	This study
*Bt*-*Bp*1106a-2	*bcpI_Bp_*_1106a-1_ (TC)	– (KM)		Fig. 5A	This study
*Bt*E264	*rfp* (KM)	–		Fig. 4C, 4D, 11	Anderson *et. al.* 2012
*Bt*-*Bp*1106a-1	*gfp* (KM)	–		Fig. 4C, 6, 7, 8,	This study
*Bt*-*Bp*1106a-2	*gfp* (KM)	–		Fig. 4D, 6, 7, 8, 9	This study
*Bt*E264	*gfp* (KM)	–		Fig. 6, 7, 8, 10	Garcia *et. al.* 2013
*Bt*Δ*bcpAIOB*	*gfp* (KM)	–	KM^S^	Fig, 6, 7, 8	Anderson *et. al.* 2012
*Bt*-*Bp*1106a-1	*rfp* (KM)	–		Fig. 9	This study
*Bt*-*Bp*1106a-1	*rfp* (KM)	*bcpI_Bp_*_1106a-2_ (TC)		Fig. 9	This study
*Bt*-*Bp*1106a-2	*gfp* (KM)	*bcpI_Bp_*_1106a-1_ (TC)		Fig. 9	This study
*Bt*-*Bp*1106a-2	*gfp* (KM)	*bcpI_Bt_*_E264_ (TC)		Fig. 9	This study
*Bt*-*Bp*1106a-1	*rfp* (KM)	*bcpI_Bt_*_E264_ (TC)		Fig. 10	This study
*Bt*E264	*gfp* (KM^S^)	*bcpI_Bp_*_1106a-1_ (TC)		Fig. 10	This study

1 Indicates genes that were cloned into the MCS of pUC18miniTn7-derived plasmids for insertion into the *Bt*E264 chromosome at one of the two *att*Tn7 sites. All genes inserted were placed under constitutive expression by P_S12_.

2 Indicates antibiotic resistance conferred by pUC18miniTn7-derived plasmids.

### Colony biofilm interbacterial competition

Competition assays were performed as previously described [6]. Overnight cultures were washed in LSLB. Inhibitor bacteria were mixed with target bacteria in a 1∶1 ratio (unless otherwise noted) at an OD_600_ of 0.2 and 20 µl of that cell suspension was deposited onto Low Salt Lysogeny Broth (LSLB) agar without antibiotic selection and incubated at 25°C for 24 hours. Bacteria were picked from the center and edge of the colony biofilm with a sterile pipette tip, suspended in PBS, diluted and plated on LSLB agar containing appropriate antibiotics to distinguish the two strains. The competitive index (C.I.) was calculated as the log of the ratio of inhibitor bacteria to target bacteria at 24 hours divided by the ratio of inhibitor bacteria to target bacteria at the start of the experiment. Two to three independent experiments were performed in triplicate.

Confocal microscopy – bacteria in colony biofilms on agar were cut out from petri dishes (agar included) and placed on a glass slide. Cover slips were added to the top of the colony biofilms. Bacteria were imaged on a Ziess 700 confocal laser scanning microscope, 63× objective with oil immersion.

### Static biofilm

Biofilm assays were performed as previously described [13]. Overnight cultures were washed in M63 medium and inoculated to an OD_600_ of 0.02 (or 0.2 for the “high inoculum”) in 400 µl M63 in chambered coverglass dishes (Thermo Scientific). For mixed strain biofilms, GFP- and RFP-producing strains were mixed at a 1∶1 ratio and inoculated to an OD_600_ of 0.02 as described above. Biofilms were incubated in humidified chambers at 37°C for 6–72 hours, washed 4–5 times with 400 µl PBS, overlaid with 400 µl PBS, and imaged by confocal laser scanning microscopy with a Zeiss LSM 700 using a 20× objective lens (low magnification) or a 63× objective lens (high magnification) with oil immersion. Z stacks were processed with Imaris ×64 v7.5.2 (Bitplane Scientific Software) and analyzed with COMSTAT [31]. Two to four representative images from two to three independent experiments were used for all analyses.

### Biofilm invasion assay

Overnight cultures of “established” strains were washed in M63 medium and inoculated to an OD_600_ of 0.2 for *Bt*::*Bp*1106a-1::*bcpI_Bt_*_E624_ and 0.02 for *Bt*E264 and allowed to incubated for 24 hours as described above, followed by 4–5 PBS washes. Overnight cultures of “invading” strains were washed in M63 medium and added to the established biofilm chambers to an OD_600_ of 2.0 and incubated for 48 hours, followed by 4–5 PBS washes. Biofilms were imaged and analyzed as described above.

### Protein comparison

Protein sequences were analyzed in Vector NTI Advance 11.

## Supporting Information

Figure S1 Diagram of *bcpAIOB* allele modularity. **A**) BcpAIOB proteins from representative *B. thailandensis* and *B. pseudomallei* strains. The different colors represent the variability of BcpA-CT, BcpI, and BcpO proteins. Gray coloring indicates more conserved sequences. Group 1 and Group 2 represent predicted modularity compatibility. **B**) Diagram of functional and non-functional chimeric proteins from this study and Nikolakakis *et. al.* 2012 [5].
(TIF)

Figure S2 Growth of strains producing chimeric BcpA proteins. Wild-type E264 (gray) and chimeric strains *Bt-Bp*1106a-1 (orange), *Bt-Bp*1106a-2 (red), *Bt-Bp*1106a-3 (blue), and *Bt-Bp*1106a-3b (green) were inoculated to an OD600 = 0.04 in LSLB and incubated at 37°C with shaking. Symbols represent the mean of triplicate samples and error bars show the SEM.
(TIF)
