# Supplementary figures and images for: Kind Discrimination and Competitive Exclusion Mediated by Contact-Dependent Growth Inhibition Systems Shape Biofilm Community Structure
Source: PLoS Pathog. 2014 Apr 17;10(4):e1004076. doi: 10.1371/journal.ppat.1004076 (PMC3990724; doi:10.1371/journal.ppat.1004076)

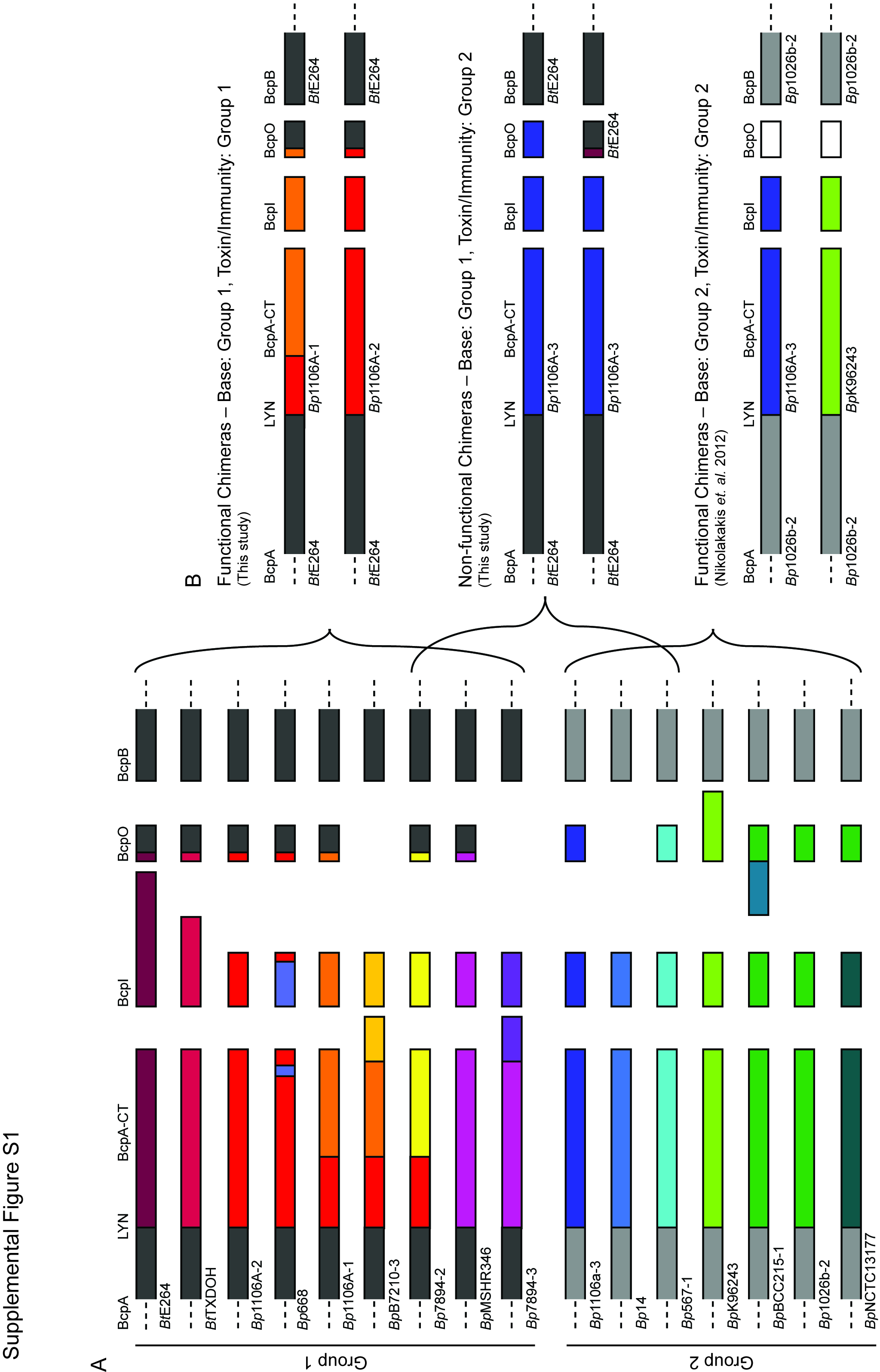

Supplement: Figure S1 — Diagram of bcpAIOB allele modularity. A) BcpAIOB proteins from representative B. thailandensis and B. pseudomallei strains. The different colors represent the variability of BcpA-CT, BcpI, and BcpO proteins. Gray coloring indicates more conserved sequences. Group 1 and Group 2 represent predicted modularity compatibility. B) Diagram of functional and non-functional chimeric proteins from this study and Nikolakakis et. al. 2012 [5]. (TIF) [file ppat.1004076.s001.tif]

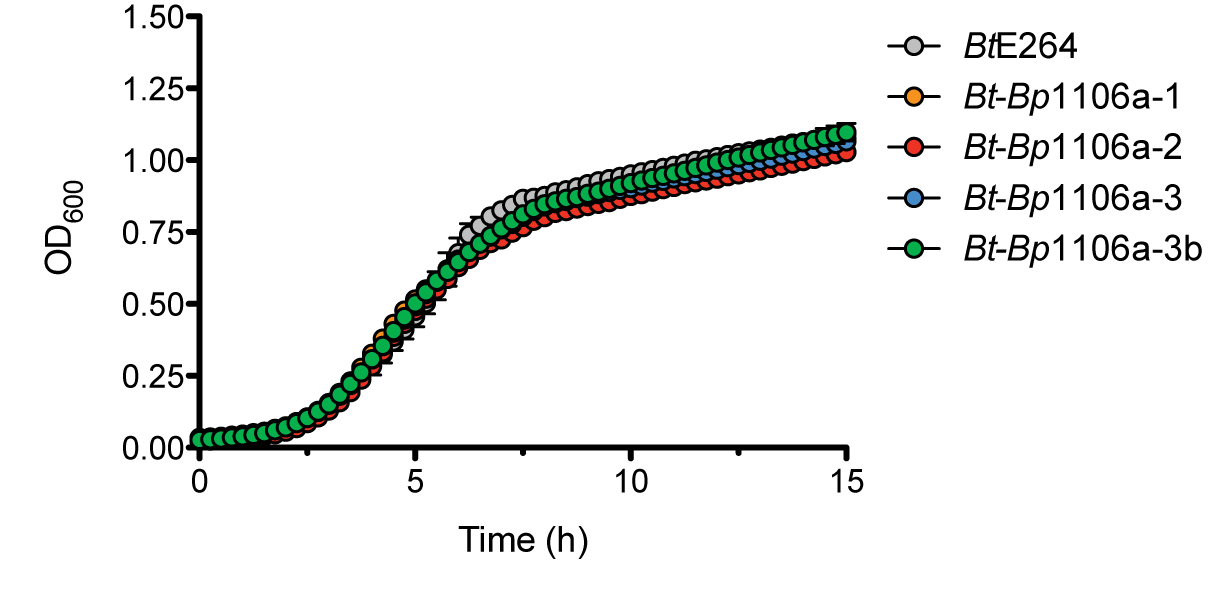

Supplement: Figure S2 — Growth of strains producing chimeric BcpA proteins. Wild-type E264 (gray) and chimeric strains Bt-Bp1106a-1 (orange), Bt-Bp1106a-2 (red), Bt-Bp1106a-3 (blue), and Bt-Bp1106a-3b (green) were inoculated to an OD600 = 0.04 in LSLB and incubated at 37°C with shaking. Symbols represent the mean of triplicate samples and error bars show the SEM. (TIF) [file ppat.1004076.s002.tif]
